# Synovial Fluid Regulates the Gene Expression of a Pattern of microRNA via the NF-κB Pathway: An In Vitro Study on Human Osteoarthritic Chondrocytes

**DOI:** 10.3390/ijms23158334

**Published:** 2022-07-28

**Authors:** Sara Cheleschi, Sara Tenti, Sauro Lorenzini, Iole Seccafico, Stefano Barbagli, Elena Frati, Antonella Fioravanti

**Affiliations:** Rheumatology Unit, Department of Medicine, Surgery and Neuroscience, Azienda Ospedaliera Universitaria Senese, Policlinico Le Scotte, 53100 Siena, Italy; sara_tenti@hotmail.it (S.T.); saurolorenz@gmail.com (S.L.); ioles388@gmail.com (I.S.); stef.bar69@gmail.com (S.B.); fratielena@unisi.it (E.F.); fioravanti7@virgilio.it (A.F.)

**Keywords:** microRNA, synovial fluid, osteoarthritis, rheumatoid arthritis, inflammation, cartilage metabolism, chondrocytes, cytokines

## Abstract

Synovial fluid (SF) represents the primary source of nutrients of articular cartilage and is implicated in maintaining cartilage metabolism. We investigated the effects of SF, from patients with osteoarthritis (OA), rheumatoid arthritis (RA), and controls, on a pattern of microRNA (miRNA) in human OA chondrocytes. Cells were stimulated with 50% or 100% SF for 24 h and 48 h. Apoptosis and superoxide anion production were detected by cytometry; miRNA (34a, 146a, 155, 181a), cytokines, metalloproteinases (MMPs), type II collagen (Col2a1), antioxidant enzymes, B-cell lymphoma (BCL)2, and nuclear factor (NF)-κB by real-time PCR. The implication of the NF-κB pathway was assessed by the use of NF-κB inhibitor (BAY-11-7082). RA and OA SF up-regulated miR-34a, -146a, -155, -181a, interleukin (IL)-1β, IL-6, tumor necrosis factor (TNF)-α, MMP-1, MMP-13, and ADAMTs-5 gene expression, while it down-regulated Col2a1. Pathological SF also induced apoptosis, reduced viability, and decreased BCL2 mRNA, whereas it increased superoxide anions, the expression of antioxidant enzymes, p65 and p50 NF-κB. Opposite and positive results were obtained with 100% control SF. Pre-incubation with BAY-11-7082 counteracted SF effects on miRNA. We highlight the role of the SF microenvironment in regulating some miRNA involved in inflammation and cartilage degradation during OA and RA, via the NF-κB pathway.

## 1. Introduction

Osteoarthritis (OA) is the most common chronic and degenerative musculoskeletal disorder and the leading cause of disability and impairment in middle-aged and older individuals [1,2]. The disease involves the entire joint and is characterized by progressive cartilage degradation, osteophytes formation, and synovial inflammation [3,4]. Rheumatoid arthritis (RA) is considered a chronic systemic, inflammatory, and progressive disease; it affects 1% of individuals aged 20–40 years worldwide, causing lifelong disability [5,6,7]. The etiology of RA is multifactorial and remains largely unknown; the disorder primarily involves the joints, leading to marked synovial inflammation, cartilage destruction, and bone erosion [8,9].

Progressive cartilage degradation and loss of extracellular matrix (ECM) elements, together with different grades of inflammation, are important hallmarks of both OA and RA diseases.

Synovial fluid (SF) represents the primary source of joint lubricants and nutrients of articular cartilage, and it is mainly implicated in the maintenance of cartilage metabolism and homeostasis. Inflammatory and degenerative processes occurring in OA and RA induced modification in the molecular and cellular composition of SF. Indeed, in these conditions, it becomes less viscous and loses its unique viscoelastic properties due to a reduction in the molecular weight and concentration of hyaluronic acid (HA), as well as due to changes in cytokines, enzymes profiles, and inflammatory white blood cells (WBCs) concentrations [10,11,12,13,14]. In particular, in RA and OA, the SF is characterized by an increased content of inflammatory mediators (prostaglandin E2, leukotriene B4), cytokines (interleukin (IL)-1β, IL-6, and tumor necrosis factor (TNF)-α), nitric oxide (NO), and reactive oxygen species (ROS), released from chondrocytes, synoviocytes, and WBCs, which contributes to articular cartilage degradation [3,5,8,15,16].

At present, only a few studies have investigated the biological effects of SF on cartilage metabolism, showing that SF from RA and OA patients stimulated pro-inflammatory and catabolic processes in human primary chondrocytes [17,18,19,20,21], while SF taken from human healthy joints seemed to have a chondroprotective role [22,23,24]. However, the results remain still limited and need to be further elucidated.

To the best of our knowledge, there is no evidence reporting the biological activities of SF on the microRNA (miRNA) expression profile. MiRNA are a class of small non-coding RNAs molecules, around 22 nucleotides long, which regulate gene expression by binding specific sequences within target messenger RNA (mRNA). A large number of evidence demonstrated that some miRNA are differentially expressed in OA cartilage samples and synovial tissue of OA and RA patients, confirming their role in the development and progression of these pathological conditions [25,26,27]. Some of the dysregulated miRNA target genes encoding for proteins implicated in extracellular matrix remodeling, pro-inflammatory activities, and redox balance in OA chondrocytes and RA fibroblast-like synoviocytes [27,28,29].

In the present study, we investigated the biological effects of SF taken from patients with OA or RA compared with control SF, in the regulation of some miRNA, cartilage metabolism, and inflammation in human primary OA chondrocytes. In this regard, we analyzed cell viability, the ratio of apoptosis, and the gene expression of miR-34a, miR-146a, miR-155, and miR-181a, the main matrix-degrading enzymes and type II collagen alpha 1 chain (Col2a1), and a pattern of cytokines. In addition, the production of mitochondrial superoxide anion and the mRNA levels of antioxidant enzymes were also evaluated. Finally, the possible mechanism underlying SF effects was assessed by analysis of the nuclear factor (NF)-κB pathway.

## 2. Results

### 2.1. SF Modulates Chondrocytes Viability and Apoptosis

**Figure 1:**
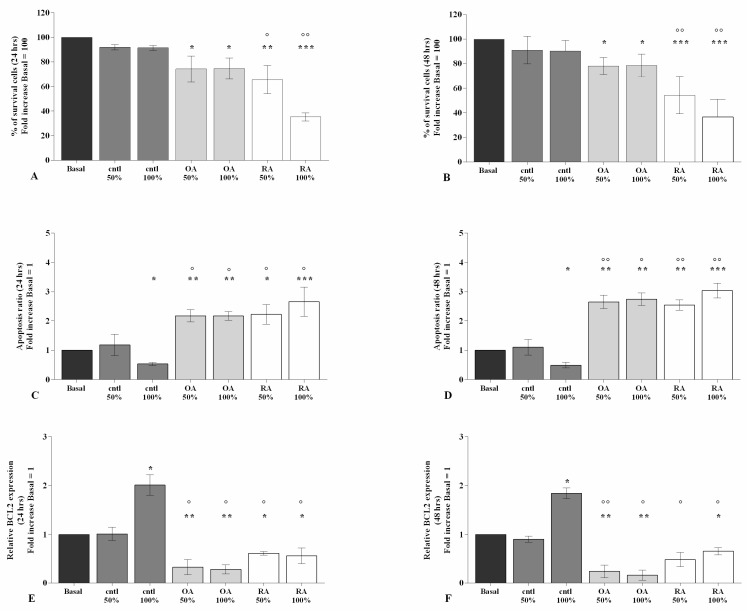
SF regulates viability and apoptosis. Human osteoarthritic (OA) chondrocytes were incubated for 24 h and 48 h with 50% and 100% SF derived from patients with OA, rheumatoid arthritis (RA), or controls. (**A**,**B**) Evaluation of cell viability by MTT assay. (**C**,**D**) Apoptosis detection performed by flow cytometry analysis and measured with annexin Alexa fluor 488 assay. Data were expressed as the percentage of positive cells for annexin-V and propidium iodide (PI) staining. (**E**,**F**) Expression levels of B-cell lymphoma (*BCL2*) analyzed by quantitative real-time PCR. The percentage of survival cells, the ratio of apoptosis, and the gene expression were referenced to the ratio of the value of interest and the value of basal condition, reported equal to 100 or 1. Data were represented as mean ± SD of triplicate values. * *p* < 0.05, ** *p* < 0.01, *** *p* < 0.001 versus basal condition. ° *p* < 0.05, °° *p* < 0.01 versus control SF.

Figure 1 and Figure S1 show the effects of the stimulus of 50% or 100% SF taken from patients with OA, RA, and controls, for 24 h and 48 h, on cell viability and apoptosis ratio evaluated by MTT assay and flow cytometry.

After 24 h and 48 h of stimulus of the cells with 50% or 100% OA SF, the percentage of survival (*p* < 0.05) and the gene levels of *BCL2* (*p* < 0.01) were significantly reduced, whereas an increase in apoptosis ratio (*p* < 0.01) was observed. Similar results were found when our cultures were stimulated with 50% or 100% RA SF (*p* < 0.05, *p* < 0.01, *p* < 0.001) (Figure 1A–F). Furthermore, viability and apoptosis were significantly affected by the stimulus with 50% or 100% OA and RA SF with respect to control SF (*p* < 0.05, *p* < 0.01); no changes were found when OA SF was compared with RA SF (Figure 1A–F).

Surprisingly, the incubation of the cells with control SF showed a significant up-regulation of the gene expression of the anti-apoptotic marker *BCL2* (*p* < 0.05), along with a decrease in apoptosis (*p* < 0.05), when tested at 100% concentration, in comparison to the basal condition (Figure 1A–F).

### 2.2. SF Regulates Inflammation and Chondrocyte Metabolism

**Figure 2:**
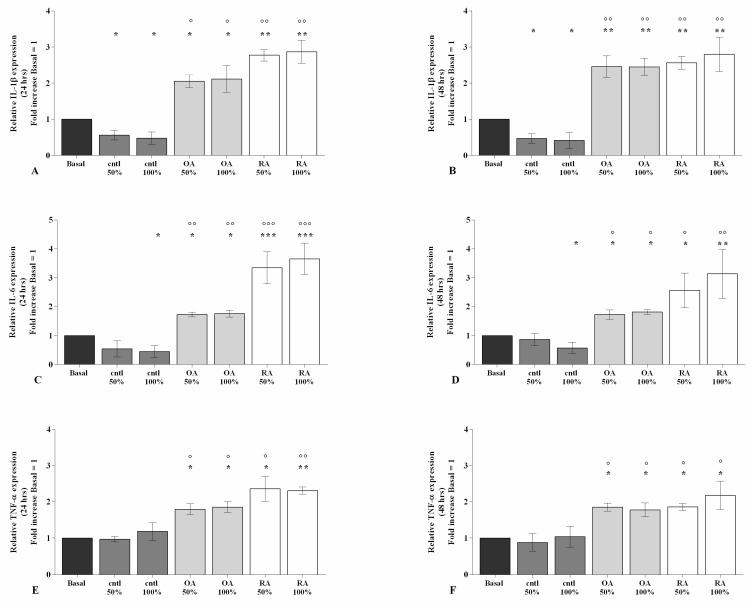
SF regulation on inflammation. Human osteoarthritic (OA) chondrocytes were incubated for 24 h and 48 h with 50% and 100% SF derived from patients with OA, rheumatoid arthritis (RA), or controls. (**A**–**F**) Expression levels of interleukin (*IL*)*-1β*, *IL-6*, and tumor necrosis factor (*TNF*)*-α* analyzed by quantitative real-time PCR. The gene expression was referenced to the ratio of the value of interest and the value of basal condition, reported equal to 1. Data were represented as mean ± SD of triplicate values. * *p* < 0.05, ** *p* < 0.01, *** *p* < 0.001 versus basal condition. ° *p* < 0.05, °° *p* < 0.01, °°° *p* < 0.001 versus control SF.

The stimulus of chondrocytes with 50% or 100% OA and RASF significantly increased the gene expression of *IL-1β*, *IL-6*, and *TNF-α* (*p* < 0.05, *p* < 0.01, *p* < 0.001), at both time-points, in comparison to the basal condition (Figure 2A–F). This effect was also reported when OA and RA SF were compared with control SF (*p* < 0.05, *p* < 0.01) (Figure 2A–F), while no modifications of the pathological SF on cytokine regulation were observed.

On the contrary, when the cells were incubated with control SF, a significant down-regulation of the transcriptional levels of *IL-1β* at both 50% and 100% concentrations (*p* < 0.05), as well as that of *IL-6* when tested at 100% concentration (*p* < 0.05), were found in comparison to the baseline (Figure 2A–F).

**Figure 3:**
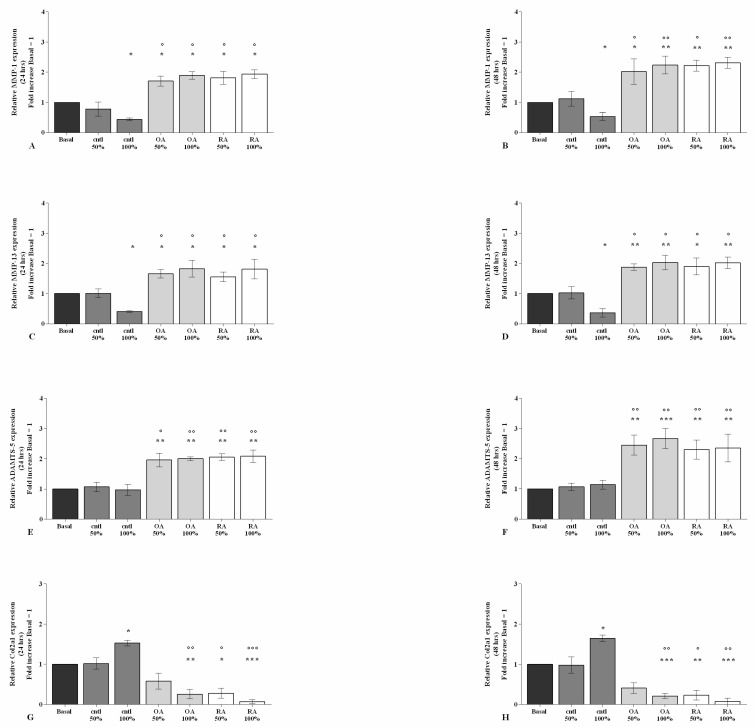
SF regulates chondrocyte metabolism. Human osteoarthritic (OA) chondrocytes were incubated for 24 h and 48 h with 50% and 100% SF derived from patients with OA, rheumatoid arthritis (RA), or controls. (**A**–**H**) Expression levels of metalloproteinase (*MMP*)*-1*, *-13*, metalloproteinase with thrombospondin motif (*ADAMTS-5*), and type II collagen (*Col2a1*) analyzed by quantitative real-time PCR. The gene expression was referenced to the ratio of the value of interest and the value of basal condition, reported equal to 1. Data were represented as mean ± SD of triplicate values. * *p* < 0.05, ** *p* < 0.01, *** *p* < 0.001 versus basal condition. ° *p* < 0.05, °° *p* < 0.01, °°° *p* < 0.001 versus control SF.

To examine the maintenance of cartilage integrity and structure under SF condition, the main matrix degrading enzymes and matrix components were tested using real-time PCR (Figure 3).

The incubation of chondrocytes with 100% control SF demonstrated a significant reduction in *MMP-1* and *MMP-13* (*p* < 0.05) and an increase in *Col2a1* (*p* < 0.05) gene expression compared to basal condition; no detectable changes were observed for *ADAMTS-5* (Figure 3A–H). On the other hand, 24 h and 48 h of stimulus with 50% or 100% OA SF significantly increased the gene levels of *MMP-1*, *MMP-13*, and *ADAMTS-5* (*p* < 0.05, *p* < 0.01), whereas it decreased the levels of *Col2a1* (*p* < 0.01, *p* < 0.001) in comparison to the baseline; 50% or 100% RA SF induced similar effects on the expression of matrix degrading enzymes and *Col2a1* (*p* < 0.05, *p* < 0.01, *p* < 0.001) (Figure 3A–H). In addition, OA and RA SF significantly affected the regulation of cartilage turnover when compared to control SF (*p* < 0.05, *p* < 0.01). Conversely, no differences were found when compared to each other (Figure 3A–F).

### 2.3. SF Affects Oxidative Stress Balance

**Figure 4:**
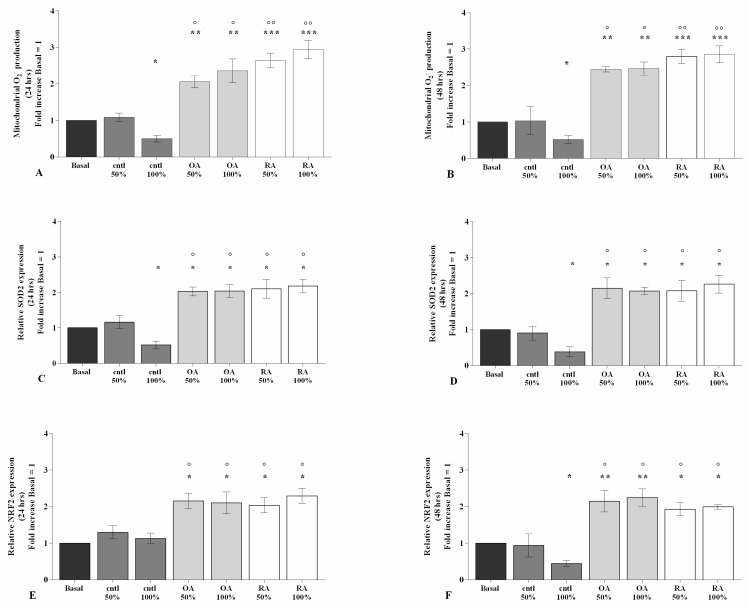
SF modulates oxidative stress balance. Human osteoarthritic (OA) chondrocytes were incubated for 24 h and 48 h with 50% and 100% SF derived from patients with OA, rheumatoid arthritis (RA), or controls. (**A**,**B**) Mitochondrial superoxide anion production evaluated by MitoSox Red staining at flow cytometry. (**C**–**F**) Expression levels of superoxide dismutase (*SOD*)*-2* and nuclear factor erythroid-derived 2-like 2 (*NRF2*) analyzed by quantitative real-time PCR. The production of superoxide anion and the gene expression were referenced to the ratio of the value of interest and the value of basal condition, reported equal to 1. Data were represented as mean ± SD of triplicate values. * *p* < 0.05, ** *p* < 0.01, *** *p* < 0.001 versus basal condition. ° *p* < 0.05, °° *p* < 0.01 versus control SF.

The potential effect of SF on the regulation of oxidant/antioxidant balance is reported in Figure 4 and Figure S2.

Flow cytometry and PCR analyses demonstrated that 100% control SF significantly limited the production of mitochondrial superoxide anion (*p* < 0.05) and the gene expression of the antioxidant enzyme *SOD-2* (*p* < 0.05), at both 24 h and 48 h of stimulus, and of transcriptional factor *NRF2* (*p* < 0.05) at 48 h, with respect to basal condition (Figure 4A–F). On the contrary, 50% or 100% OA and RA SF induced a significant increase in superoxide anion production (*p* < 0.01, *p* < 0.001), as well as in the gene expression of *SOD-2* (*p* < 0.05, *p* < 0.01) and *NRF2* (*p* < 0.05, *p* < 0.01) gene expression (Figure 4A–F). OA and RA SF also promoted the oxidative stress status when compared to control SF, at both analyzed time points (*p* < 0.05, *p* < 0.01), with no differences between them (Figure 4A–F).

### 2.4. SF Regulates the Gene Expression of a Pattern of miRNA

**Figure 5:**
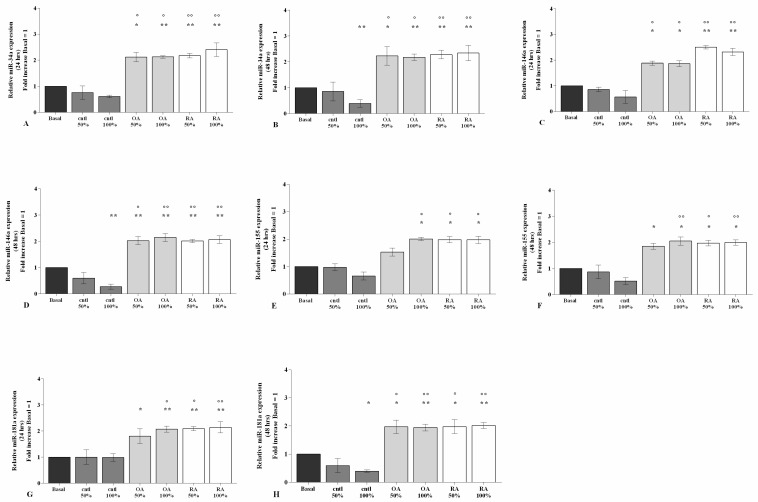
SF regulation on miRNA. Human osteoarthritic (OA) chondrocytes were incubated for 24 h and 48 h with 50% and 100% SF derived from patients with OA, rheumatoid arthritis (RA), or controls. (**A**–**H**) Expression levels of microRNA (*miR*)*-34a*, *miR-146a*, *miR-155*, and *miR-181a* analyzed by quantitative real-time PCR. The gene expression was referenced to the ratio of the value of interest and the value of basal condition, reported equal to 1. Data were represented as mean ± SD of triplicate values. * *p* < 0.05, ** *p* < 0.01 versus basal condition. ° *p* < 0.05, °° *p* < 0.01 versus control SF.

The gene expressions of *miR-34a*, *miR-146a*, *miR-155*, and *miR-181a*, evaluated by real-time PCR, were significantly increased (*p* < 0.05, *p* < 0.01), at both 24 h and 48 h, in our cell cultures stimulated with 50% or 100% of OA or RA SF when compared to the baseline (*p* < 0.05, *p* < 0.01) (Figure 5A–H). A similar trend was also maintained when OA and RA SF were compared with control SF (*p* < 0.05, *p* < 0.01) (Figure 5A–H). No differences between OA and RA SF on miRNA regulation were observed.

Surprisingly, 48 h of treatment with control SF significantly reduced the levels of *miR-34a*, *miR-146a*, and *miR-181a* when tested at 100% concentration (*p* < 0.05) with respect to the baseline (Figure 5A–H).

### 2.5. SF Induces NF-κB Pathway Activation

**Figure 6:**
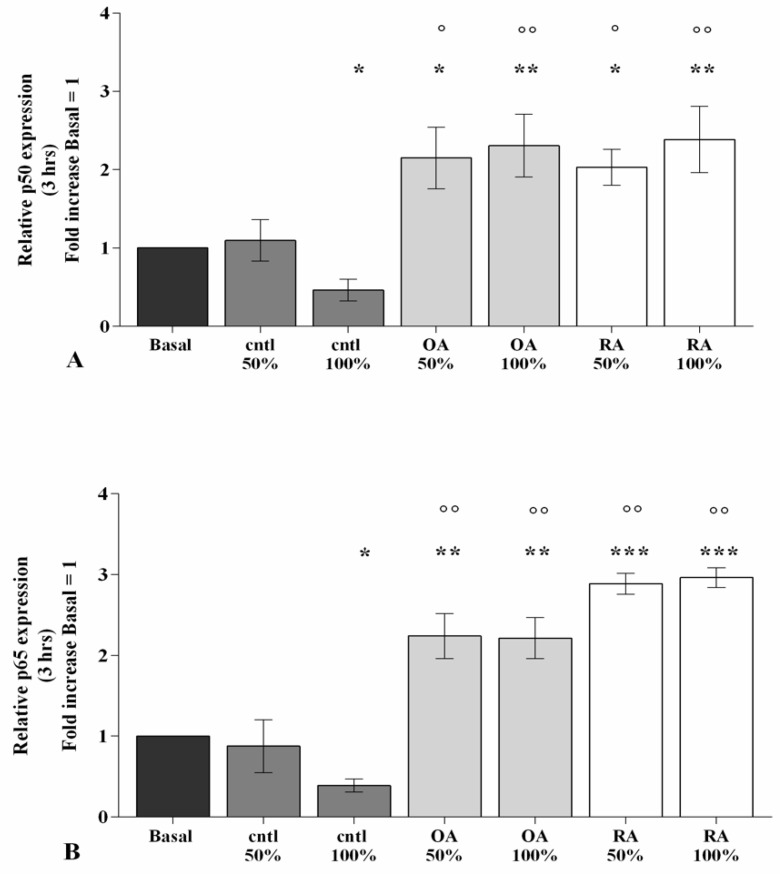
SF modulates the NF-κB signaling pathway. Human osteoarthritic (OA) chondrocytes were incubated for 3 h with 50% and 100% SF derived from patients with OA, rheumatoid arthritis (RA), or controls. (**A**,**B**) Expression levels of *p50* and *p65* subunits analyzed by quantitative real-time PCR. The gene expression was referenced to the ratio of the value of interest and the value of the basal condition, reported equal to 1. Data were represented as mean ± SD of triplicate values. * *p* < 0.05, ** *p* < 0.01, *** *p* < 0.001 versus basal condition. ° *p* < 0.05, °° *p* < 0.01 versus control SF.

Figure 6 reports the effect of SF on the regulation of the NF-κB signaling pathway after 3 h of stimulating OA chondrocytes with 50% or 100% SF from OA, RA, and controls. The real-time PCR analysis showed a significant reduction in *p50* and *p65* subunits gene expression after incubation with 100% control SF in comparison to the basal condition (Figure 6A,B). Conversely, 50% or 100% OA and RA SF significantly up-regulated the transcriptional levels of both subunits (*p* < 0.05, *p* < 0.01, *p* < 0.001), with respect to the baseline, revealing a particular exacerbation of *p65*. OA and RA SF stimulus was also effective when compared to control SF (*p* < 0.05, *p* < 0.01), while no modifications were observed among the pathological SF samples (Figure 6A,B).

### 2.6. SF Regulates miRNA Expression via the NF-κB Pathway

**Figure 7:**
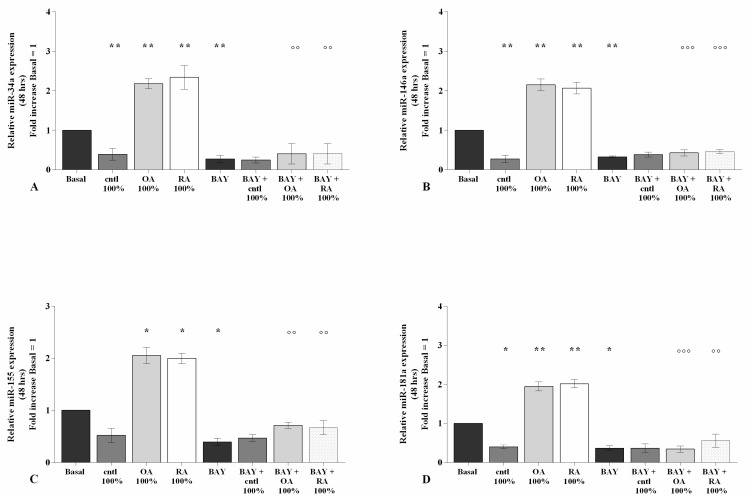
SF modulates the NF-κB pathway. Human osteoarthritic (OA) chondrocytes were pre-incubated for 3 h with a specific nuclear factor (NF)-κB inhibitor (BAY 11-7082, IKKα/β, 1 μM), and then for 48 h with 100% SF derived from patients with OA, rheumatoid arthritis (RA), or controls. (**A**–**D**) Expression levels of microRNA (*miR*)*-34a*, *miR-146a*, *miR-155*, and *miR-181a* analyzed by quantitative real-time PCR. The gene expression was referenced to the ratio of the value of interest and the value of basal condition, reported equal to 1. Data were represented as mean ± SD of triplicate values. * *p* < 0.05, ** *p* < 0.01, versus basal condition. °° *p* < 0.01, °°° *p* < 0.001 versus control SF.

The involvement of the NF-κB pathway in mediating SF-induced effects on miRNA expression has been assessed using a specific NF-κB inhibitor (IKKα/β, BAY 11-7082) (Figure 7).

Real-time PCR analysis revealed that the gene expression of the studied miRNA was significantly decreased (*p* < 0.05, *p* < 0.01) in OA chondrocytes incubated with BAY 11-7082 in comparison to the basal condition (Figure 7A–D). The co-treatment of the cells with BAY 11-7082 and 100% OA and RA SF did not show any change in miRNA expression with respect to chondrocytes incubated with BAY 11-7082 alone (Figure 7A–D).

Furthermore, the presence of the NF-κB inhibitor significantly limited the effect of OA and RA SF on the expression levels of the analyzed miRNA (*p* < 0.05, *p* < 0.01). No modifications with 100% control SF were observed (Figure 7A–D).

## 3. Discussion

MiRNA play an important role in the pathogenesis of different musculoskeletal diseases, including OA and RA [30]. Data suggest that miRNA have both beneficial and detrimental effects on the joints. In fact, a lot of studies proved their activity in cartilage metabolism, as well as in pathological processes including inflammation, apoptosis, and oxidative stress [25,30].

SF is mainly involved in the maintenance and health of the articular cartilage. It has been observed that chondrocyte metabolism is deeply influenced by the specific composition of SF [12,14]. This evidence stimulated an increasing interest in comprehending the exact effect of the SF microenvironment in cartilage homeostasis.

In this study, we examined the effects of OA and RA SF, tested at 50% and 100%, in a pattern of miRNA associated to the pathogenesis of OA and RA. The possible implication of the NF-κB pathway was also assessed. Furthermore, we evaluated the effects of the OA and RA SF on inflammation, apoptosis, and oxidative stress. SF from patients with knee injuries who underwent anterior cruciate ligament surgery was used as a control.

First of all, our data confirmed a significant increase in the gene expression of the main pro-inflammatory cytokines *IL-1β*, *IL-6*, and *TNF-α* in OA chondrocytes incubated with RA SF for 24 h and 48 h. Similarly, SF from patients with OA showed the same regulation of the studied cytokines. These results are in agreement with previous studies. In particular, some authors demonstrated a relevant increase in *IL-1β*, *IL-6*, *IL-8*, and *TNF-α* expression in human primary chondrocytes and mesenchymal stem cells treated with OA or RA SF, for 24 h and 48 h [28,29,31]. Furthermore, the stimulus of our cells derived from OA and RA SF determined a marked increase in the gene expression of the main matrix-degrading enzymes *MMP-1*, *MMP-13*, and *ADAMTS-5*, while a down-regulation of Col2a1 was observed. In a similar manner, Carballo et al. [21] found raised gene levels of *MMP-3*, *MMP-13*, *ADAMTS-5*, and *IL-18* in human primary chondrocytes stimulated with OA SF for 24 h and 72 h. These findings suggest the contribution of SF to the ECM-degrading processes occurring over the course of this disease.

The elevated levels of IL-1β, IL-6, and TNF-α, as well as of MMPs, found in the SF of both OA and RA patients confirmed their important role in the pathogenesis of these joint diseases [12,32]. Therefore, an excess of these pro-inflammatory mediators and enzymes in SF can affect chondrocyte homeostasis and induce the production of other cytokines and matrix-degrading enzymes; in addition, they also inhibit the synthesis of proteoglycans and type II collagen, thereby causing cartilage degradation in OA and RA [8,32].

Surprisingly, when our chondrocytes were incubated with 50% or 100% control SF, at both analyzed time points, a significant reduction in the gene expression of *IL-1β* and *IL-6* was observed, as well as of *MMP-1* and *MMP-13*, while an over-expression of *Col2a1* was found. These results are partially consistent with a study performed on bovine cartilage explant cultures incubated with different SF percentages isolated from healthy bovine joints, for 12 days, demonstrating the anabolic and anti-catabolic activities of the SF used [33].

The regulation of chondrocyte survival is important for the maintenance of proper cartilage structure and function. Apoptosis is an active, physiological process of cell death, playing a critical role in retaining the homeostasis of tissues and cells [34]; it is regulated by various genes, such as those of the *BCL-2* family, identified as controllers and inductors of this mechanism [35]. Dysregulation of apoptosis is, thus, associated with a variety of diseases including inflammatory and degenerative disorders such as OA and RA [35,36].

Cell viability and apoptosis analyses performed in the present study showed a reduction in survival and an increase in apoptotic ratio, with a concomitant down-regulation of the anti-apoptotic marker *BCL2*, in OA chondrocytes stimulated with 50% and 100% RA or OA SF. These data confirm what was found in previous studies. In 2007, Schuerwegh et al. [18] observed a significant decrease in cell viability and cell proliferation, along with an increase in apoptosis, in bovine articular chondrocytes incubated for 48 h with SF derived from patients with RA. Later, a group of investigators reported that the number of vital cells appeared clearly reduced after treatment of human OA chondrocytes with RA or OA SF, from 24 h to 72 h; conversely a strong induction of early apoptosis was observed in a time-dependent manner [19,20]. These authors pointed out that the highest effect of pathological SF on cell survival was achieved after 24 h of treatment. During further incubation (until 72 h), the number of vital cells remained stable, suggesting a sort of adaptation of chondrocytes to the surrounding microenvironment after initial damage. This hypothesis could be in line with our results considering that we observed SF effects at 24 h of stimulus, and a similar trend, without substantial differences, was maintained at 48 h.

Interestingly, to the best of our knowledge, we demonstrated for the first time the anti-apoptotic activity of control SF, at 100% concentrations at both tested time points.

In recent years, the integrity of the mitochondrial structure has been considered a prerequisite for normal chondrocyte survival. Degenerative factors and inflammatory mediators, such as cytokines, induce defects in mitochondrial function and lead to excessive ROS generation; this condition contributes to cartilage degradation and synovial inflammation [3,37]. Accumulation of ROS in the joint, with a concomitant failure of the antioxidant scavenging system, progressively inhibits the synthesis of glycosaminoglycans and type II collagen fibers, induces apoptosis, and activates the production of matrix-degrading enzymes [3,37]. In addition, ROS overproduction exacerbates synovitis and leads to the release of pro-inflammatory cytokines, in turn, stimulating the synthesis of new ROS [37,38].

In this study, the analysis of oxidative stress showed an overproduction of mitochondrial superoxide anions in OA chondrocytes treated with 50% or 100% RA or OA SF. Pathological SF also increased the gene expression of antioxidant enzyme, *SOD2*, and the transcriptional factor *NRF2.* This is the first experience revealing the involvement of SF components in the regulation of redox balance. However, a study by Schuerwegh and collaborators [18] seems to partially sustain our results, albeit not comparable; the authors reported a significant increase in NO production after treatment of bovine articular chondrocytes with RA SF, for a period of 48 h.

Furthermore, Sun at el. [33] demonstrated the ability of 1% normal SF to reduce the production of NO, induced by IL-1β, in bovine articular cartilage explants; this activity was effective in a time-dependent manner, until 12 days of incubation. These data appear in contrast with our findings since we did not observe any substantial effect of control SF in regulating oxidative stress. We hypothesize that this could be ascribable to the different experimental procedures employed, such as the samples used, the parameters analyzed, and the treatment conditions applied.

The increase in antioxidant agents observed after stimulus of chondrocytes with pathological SF, concomitant with excessive ROS production, could be explained as an acute adaptive mechanism of the cell against the overproduction of free radicals. We speculate that, in response to continuous and cumulative oxidative stress status, a protective increase in ROS scavenging activity can be induced through the activation of transcription factor *NRF2* [28,38,39,40], as confirmed by various authors [41,42,43].

In the present study, for the first time, we investigated the potential effect of SF in the regulation of some miRNA associated to the joint damage occurring in OA and RA.

In particular, miR-34a has been known to be implicated in activating apoptosis signaling, limiting cell proliferation, and modulating redox balance in human OA chondrocytes and synovial fibroblasts [28,29,43,44,45]. In addition, miR-146a, traditionally identified as a regulator of inflammation, has been recently recognized as an activator of apoptosis and oxidative stress by its direct effect on SMAD4 and NRF2 transcriptional factors, in OA cells [28,29,46]. Similarly, miR-181a activates apoptosis and oxidative stress by targeting the anti-apoptotic marker BCL2 and modulating the oxidant/antioxidant system in different cell types [28,47,48]. Furthermore, it is a regulator of B and T cell development and stimulates the production of pro-inflammatory cytokines in RA synovial fibroblasts [49]. MiR-155 has been primarily associated to the maturation and activation of innate and adaptive immune system cells, and also found highly expressed in synovial fluid of RA patients [50]. The described properties of these miRNA make them very suitable candidates for regulating inflammatory and degenerative articular processes [25,30]. Thus, we explored the possible influence of pathological SF on their expression profile in chondrocyte cultures to provide new information on the complex mechanisms underlying OA and RA.

Interestingly, for the first one, we demonstrated the up-regulation of miR-34a, miR-146a, miR-155 and miR-181a gene expression induced by OA and RA SF, while opposite effects were observed when the cells were incubated with 100% control SF. These data partially sustained the results on miR-155 obtained by Li et al. in PBMC-derivative macrophages incubated with OA SF for 24 h and 48 h [51]. To the best of our knowledge, no data from the literature are available about the regulation of SF on the other studied miRNA.

Lastly, we supposed that the observed effects induced by SF can be associated to the regulation of the NF-κB signaling pathway.

It is well known that NF-κB signaling represents one of the most prominent mechanisms activated in the pathogenesis of inflammation-related diseases, such as OA and RA [52,53]. Indeed, in these pathological conditions, NF-κB is highly expressed in articular cartilage and synovial tissue [52,53,54]. It is triggered by pro-inflammatory cytokines, such as IL-1β, and ECM degradation products, and the activated NF-κB modulates the expression of several cytokines, chemokines, and matrix-degrading enzymes; all these features accelerate catabolic events responsible for promoting cartilage degradation and inflammation [55,56]. Moreover, the direct involvement of the NF-κB pathway on miRNA-related post-transcriptional regulation of inflammation, apoptosis, and oxidative stress in OA chondrocytes and synovial fibroblasts has been reported [28,29,57,58,59].

In this experience, we found an increase in the gene expression of the main NF-κB subunits, *p50* and *p65*, after stimulus of OA chondrocytes, for 3 h, with 50% or 100% RA and OA SF, while their reduction in the presence of 100% control SF was observed. The results obtained by Sayegh et al. [31] are in line with our outcomes. In particular, they revealed that pro-inflammatory RA SF, added to the culture medium of mesenchymal stem cells, was able to significantly inhibit the protein expression of NF-κB inhibitor, IκB, resulting in a consequent activation of the pathway; the authors assumed that the presence of different cytokines in SF, including TNF-α, was essential to regulate the activation of the signaling pathway, through the TNF/NF-κB axis.

Our results also confirmed the regulation of miRNA by NF-κB, as shown by the use of a specific NF-κB inhibitor, BAY11-7082, according to previous observations [28,29,57,58,59]. Furthermore, we proved, for the first time, that the presence of BAY11-7082 in our cultures, down-regulating NF-κB, reduced the effect of SF on miR-34a, miR-146a, miR-155, and miR-181a gene expression.

Taken together, our results suggest that a pathological SF, abundant in pro-inflammatory mediators and degrading factors, can induce the expression of some miRNA; miRNAs, in turn, have an active role on inflammation, cartilage turnover, apoptosis and oxidative stress, through the activation of the NF-κB pathway, triggering a vicious circle (Figure S3).

Otherwise, the observed protective effects of control SF can be attributed to low content of cytokines and other inflammatory mediators, and to a high amount of high-molecular-weight HA [60]. High-molecular-weight HA has demonstrated to have potent anti-inflammatory effects in synovial fibroblasts, reducing NF-κB activation and IL-1β, IL-6, and TNF-α production [61,62,63].

## 4. Materials and Methods

### 4.1. Synovial Fluid Collection

SF was obtained from the knees of four patients with OA (two females and two males, with age ranging from 62 to 70 years) and four patients suffering from RA (three females and one male, with age ranging from 55 to 59 years). Diagnoses of knee OA and RA were ensured by ACR/EULAR classification criteria [64,65] (Table S1). All patients were followed in our Rheumatology Unit (Azienda Ospedaliera Universitaria Senese, Siena, Italy) and underwent knee aspirations for diagnostic evaluation or prior to an intra-articular glucocorticoid or hyaluronic acid injection. Four healthy subjects (one female and three males, with age ranging from 30 to 35 years) with knee injuries who underwent anterior cruciate ligament reconstruction under spinal anesthesia were used as controls. They have no signs or symptoms related to OA, RA, or other inflammatory or degenerative joint diseases. During surgery, SF was aseptically aspirated with an 18-gauge needle. There was no evidence of contamination of the SF samples at the time of aspiration. The samples from healthy subjects were provided by the Orthopedic Surgery Unit (University of Siena, Italy).

The participants presented no clinical or laboratory signs of infection and gave their informed consent prior to SF collection.

SF was put in a sterile container and stored in a refrigerator at 4 °C until it was examined within 24 h of joint aspiration. SF samples were analyzed by an expert examiner on site, blinded to the clinical findings of the subjects. After the first macroscopic analysis, SF was microscopically examined under optical light microscopy for total and differential WBC count using a Bürker counting chamber and pre-stained slides for cell morphology [66,67]. A crystal search was performed using polarized compensated light microscopy, and a negative result was necessary before using SF for the study [66,67].

Following the analysis, SF samples were diluted to 20% in DMEM with 10% FBS, due to their high viscosity, and then filtered through a 70-μm nylon mesh to remove debris and prevent possible contamination of the cultures. Samples were transferred to microcentrifuge tubes, and centrifuged at 2000 rpm for 10 min before storing the supernatant at −80 °C until use.

### 4.2. Cell Cultures

Human OA articular cartilage was obtained from the femoral heads of five non-obese (BMI ranging from 20 to 23 kg/m^2^) and non-diabetic patients (two men and three women, age ranging from 65 to 75) with hip OA according to ACR criteria [64], subjected to total hip replacement surgery. OA chondrocytes were derived from the area adjacent to the OA lesion [68]. Articular cartilage was supplied by the Orthopedic Surgery Unit, University of Siena, Italy. The use of human articular specimens was allowed by the authorization of the Ethic Committee of Azienda Ospedaliera Universitaria Senese/Siena University Hospital (decision no. 13931/18), and informed consent was provided by each donor.

Chondrocytes isolation was provided immediately after surgery. In particular, cartilage fragments were aseptically dissected from each donor and processed by an enzymatic digestion, as previously described [48]. For growth and expansion, cells were cultured in Dulbecco’s modified Eagle’s medium (DMEM) (Euroclone, Milan, Italy) with phenol red and L-glutamine, supplemented with 10% fetal bovine serum (FBS) (Euroclone, Milan, Italy), 200 U/mL penicillin, and 200 µg/mL streptomycin (P/S) (Sigma-Aldrich, Milan, Italy). The medium was changed every 2–3 days, and the cell morphology was examined daily with an inverted microscope (Olympus IMT-2, Tokyo, Japan). OA primary chondrocytes at the first passage were employed for the experiments.

### 4.3. Treatment of Cell Cultures

Human OA chondrocytes were plated in six-well dishes at a starting density of 1 × 10^5^ cells/well until 85% confluence. SF samples (4 OA, 4 RA, and 4 healthy controls) were diluted to 20% in DMEM with 10% FBS, and then used for the treatment. Medium was removed from the cultures and replaced with 50% (50% SF at 20% in DMEM 10% FBS + 50% DMEM 10% FBS) or 100% (100% SF at 20% in DMEM 10% FBS) SF from patients with knee OA, RA, and controls, for a period of 24 h and 48 h.

After the treatment, the cells were recovered and immediately processed to carry out the MTT assay, flow cytometry analysis, and quantitative real-time PCR.

Some cultures were pre-incubated for 2 h with BAY 11-7082 1 μM (NF-κB inhibitor, IKKα/β, Sigma–Aldrich, Milan, Italy) and then treated with SF. Afterwards, the gene expression of the studied miRNA (*miR-34a*, miR-146a, miR-155, and miR-181a) was evaluated.

### 4.4. MTT Assay

After treatment, the viability of the cells was evaluated by a 3-(4,4-dimethylthiazol-2-yl)-2,5-diphenyl-tetrazoliumbromide (MTT) (Sigma-Aldrich, Milan, Italy) test to assess the percentage of survival cells.

Chondrocytes were grown in 12-well dishes at a starting density of 5 × 10^4^ and, after treatment, were incubated for 3 h at 37 °C in a culture medium containing 10% of 5 mg/mL of MTT (Sigma–Aldrich, Milan, Italy). Then, the medium was removed and 0.2 mL of dimethyl sulfoxide (DMSO) (Rottapharm Biotech, Monza, Italy) were added to the wells to solubilize the formazan crystals. The absorbance was measured at 570 nm in a microplate reader (BioTek Instruments, Inc., Winooski, VT, USA).

The percentage of survival cells was evaluated as follows: (absorbance of considered sample)/(absorbance of control) × 100. The obtained data were reported as optical density units per 104 adherent cells.

### 4.5. Apoptosis Detection

Apoptotic cells were evaluated using an annexin V-FITC and propidium iodide (PI) (ThermoFisher Scientific, Milan, Italy) kit. Chondrocytes were seeded in 12-well plates (8 × 10^4^ cells/well) for 24 h in DMEM with 10% FBS, before the treatment procedure. Then, the cells were washed and harvested using trypsin, collected into cytometry tubes, and centrifuged at 1500 rpm for 5 min. The supernatant was replaced, and the pellet was re-suspended in 100 μL of 1 × annexin-binding buffer, 5 μL of Alexa Fluor 488 annexin-V conjugated to fluorescein (FITC, green fluorescence), and 1 μL of 100 μg/mL PI (red fluorescence) working solution, incubated at room temperature for 15 min in the dark. Afterward, 600 μL of 1 × annexin-binding buffer was added to the tubes before analysis using a flow cytometer.

A total of 10,000 events (1 × 10^4^ cells per assay) were measured by the instrument, and the results were analyzed with Cell Quest software (Version 4.0, Becton Dickinson, San Jose, CA, USA). Apoptosis analysis was carried out considering the simultaneous staining of cells with Alexa Fluor 488 annexin-V and PI. The results were expressed as the percentage of cells positively stained by each dye (total apoptosis) [69].

### 4.6. Mitochondrial Superoxide Anion (•O^2−^) Production

OA chondrocytes were seeded into 12-well plates (8 × 10^4^ cells/well) for 24 h in DMEM with 10% FBS, before the treatment procedure. Then, the cells were incubated in PBS (Euroclone, Milan, Italy) and MitoSOX Red for 15 min at 37 °C in the dark, to evaluate mitochondrial superoxide anion (•O^2−^) production. Cells were then harvested by trypsin, collected into cytometry tubes, and centrifuged at 1500 rpm for 10 min. Cells were then dissolved in saline solution before flow cytometry analysis. A density of 1 × 10^4^ cells per assay (a total of 10,000 events) was measured by the instrument and data were analyzed with CellQuest software (Version 4.0, Becton Dickinson, San Jose, CA, USA). Results were collected as the median fluorescence (AU), represented by the mean of three independent experiments [29].

### 4.7. RNA Isolation and Quantitative Real-Time PCR

Cells were grown and maintained in six-well dishes at a starting density of 1 × 10^5^ cells/well until they became 85% confluent in DMEM supplemented with 10% FBS, before the treatment procedure. After treatment, cells were collected, and total RNA was extracted using TriPure Isolation Reagent (Euroclone, Milan, Italy), according to the manufacturer’s instructions, before storing at −80 °C. The concentration, purity, and integrity of RNA were evaluated by measuring the OD at 260 nm and the 260/280 and 260/230 ratios using a Nanodrop-1000 (Celbio, Milan, Italy).

Reverse transcription for target genes was carried out by the QuantiTect Reverse Transcription Kit (Qiagen, Germany), while for miRNA it was carried out by the cDNA miScript PCR Reverse Transcription Kit (Qiagen, Hilden, Germany), according to the manufacturer’s instructions.

Target genes and miRNA were examined by real-time PCR using QuantiFast SYBR Green PCR (Qiagen, Hilden, Germany) and miScript SYBR Green (Qiagen, Hilden, Germany) kits, respectively. A list of primers used for PCR reactions is reported in Table S2.

All qPCR reactions were achieved in glass capillaries by a LightCycler 1.0 (Roche Molecular Biochemicals, Mannheim, Germany) with LightCycler Software Version 3.5. The reaction procedure was described in detail in our previous studies [57,69].

For the data analysis, the Ct values of each sample and the efficiency of the primer set were calculated and converted into relative quantities [70,71]. The normalization was performed considering actin beta (ACTB) for target genes and Small Nucleolar RNA, C/D Box 25 (*SNORD-25*) for miRNA as the housekeeping genes [72].

### 4.8. Statistical Analysis

Three experiments were performed for each SF (4 OA, 4 RA, and 4 healthy controls), and the results were expressed as the mean ± standard deviation (SD) of triplicate values for each experiment. The normal distribution of data was evaluated by Shapiro–Wilk, D’Agostino and Pearson, and Kolmogorov–Smirnov tests. MTT, flow cytometry results, and quantitative real-time PCR, were each analyzed via a mixed model ANOVA with Bonferroni post hoc test. All analyses were carried out using the SAS System (SAS Institute Inc., Cary, NC, USA) and GraphPad Prism 6.1. A *p*-value of < 0.05 was defined as statistically significant.

## 5. Conclusions

The results of the present study highlight the potential role of SF in the regulation of a pattern of miRNA in human OA chondrocytes, via the NF-κB signaling pathway.

First of all, we observed that SF, taken from patients with RA or OA, has inflammatory and degrading activities on cartilage, the expression of cytokines, and matrix degrading enzymes, activating apoptosis and oxidative stress, as well as the NF-κB pathway, in human OA chondrocytes. Conversely, healthy SF showed opposite and positive effects.

Our data demonstrated, for the first time, the role of SF in modulating the expression levels of miR-34a, miR-146a, miR-155, and miR-181a, mainly involved in inflammation and cartilage metabolism occurring in OA and RA; moreover, the observed transcriptional modifications of miRNA seem to be due to the modulation of the NF-κB signaling pathway.

Thus, we underline that a specific composition of SF microenvironment may differently influence inflammation and chondrocyte integrity and metabolism. We suppose that this aspect is in part related to (1) the presence of pro-inflammatory cytokines and matrix degrading factors inside the fluid, (2) the number of WBCs, attesting the presence of an inflammatory state, and (3) the content of high-molecular-weight HA, lubricin, and other bioactive molecules.

Taken together, our data provide preliminary fundamentals to improve the knowledge about the relevance of SF, as well as its composition, in the regulation of cartilage homeostasis, even through miRNA regulation. Our observations can help to improve the understanding of the complex mechanisms underlying the pathogenesis of OA and RA.

However, further experiments are required to support our hypothesis, considering the difficulty of translating the results obtained from in vitro studies into clinical practice.

In addition, our study presents some limitations that have to be noticed. The main limitation is related to the discrepancy in age between the groups of patients and controls. This is due to the difficulty to find normal controls, without any sign of rheumatologic disease, in older subjects. Second, the analysis of the rheological properties of the studied SF, as well as of their specific content in pro-inflammatory mediators and HA, should provide further information useful to investigate the relationship more deeply between the different components of SF and their effects in inflammatory and degrading processes and in miRNA expression profiles. Furthermore, a multivariate analysis investigating the possible association among the different studied mediators could be useful to improve the knowledge about the pathogenetic mechanisms underlying inflammatory and/or degenerative diseases. Another important limitation is the lack of the analysis of protein expression of the evaluated target genes, to confirm whether the changes occurring at the gene expression level are also reflected in protein regulation. In addition, transfection experiments using specific miRNA inhibitors may point out the implication of these molecules in mediating SF effects.

## Data Availability

The data presented in this study are available on request from the corresponding author.

## Supplementary Materials

The following supporting information can be downloaded at: https://www.mdpi.com/article/10.3390/ijms23158334/s1.
