# Supplementary material for: Synovial Fluid Regulates the Gene Expression of a Pattern of microRNA via the NF-κB Pathway: An In Vitro Study on Human Osteoarthritic Chondrocytes
Source: Int J Mol Sci. 2022 Jul 28;23(15):8334. doi: 10.3390/ijms23158334 (PMC9369022; doi:10.3390/ijms23158334)
Supplement: Supplementary file 1 [file ijms-23-08334-s001.zip › Table S1.pdf]

Table S1. Demographic, clinical, and laboratory features of patients and healthy controls, who served as donors of SF samples.

Characteristic	**RA**	**OA**	**HC**
Patients, n	4	4	4
Age, years	56 ± 1.73	66 ± 3.36	33 ± 2.38
Gender, female/male	3/1	2/2	1/3
Duration, years	5 ± 1.70	7 ± 1.5	
**Laboratory features**			
RF, positive, n (%)	4 (100)	0 (0)	
Anti-CCP, positive, n (%)	4 (100)	0 (0)	
CRP (mg/dL)	3.75 ± 2.8	0.9 ± 0.35	
ESR (mm/h)	47 ± 9.39	27.5 ± 6.45	
**Disease activity score**			
DAS28-ESR	3.3 ± 0.3	/	
**K-L score**	/		
II		3	
III		1	
**Treatment**			
NSAIDs, n (%)	4 (100)	3 (75)	2 (50)
Acetaminophen, n (%)	1 (25)	1 (25)	2 (50)
Glucocorticoid (<10 mg prednisone/day or equivalent)	0 (0)	4 (100)	

Values are expressed as media ± standard deviation, except where indicated otherwise.

Abbreviations: RA = rheumatoid factor; OA = osteoarthritis; HC = healthy controls; RF = rheumatoid factor; Anti-CCP = anti-cyclic citrullinated peptide antibody; CRP = C-reactive protein; ESR = erythrocyte sedimentation rate; SF = synovial fluid; DAS28 = disease activity score of 28 joints; NSAIDs = non-steroidal anti-inflammatory drugs.
