# Supplementary material for: Synovial Fluid Regulates the Gene Expression of a Pattern of microRNA via the NF-κB Pathway: An In Vitro Study on Human Osteoarthritic Chondrocytes
Source: Int J Mol Sci. 2022 Jul 28;23(15):8334. doi: 10.3390/ijms23158334 (PMC9369022; doi:10.3390/ijms23158334)
Supplement: Supplementary file 1 [file ijms-23-08334-s001.zip › Table S2.pdf]

**Table S2. List of primers used for quantitative real time PCR.**	
**Genes**	**Cat. No. (Qiagen)**
IL-1β	QT00021385
IL-6	QT00083720
TNF-α	QT00029162
MMP-1	QT00014581
MMP-13	QT00001764
ADAMTS-5	QT00011088
Col2a1	QT00049518
BCL2	QT00000721
SOD-2	QT01008693
NRF2	QT00027384
NFKB1	QT00063791
RELA	QT01007370
ACTB	QT00095431
miR-34a	MS00003318
miR-146a	MS00003535
miR-155	MS00031486
miR-181a	MS00006692
SNORD-25	MS00014007

Abbreviations: *IL-1β* = interleukin 1β, *IL-6* = interleukin 6, *TNF-α* = tumor necrosis factor *α*, *MMP-1* = metalloproteinase 1, *MMP-13* = metalloproteinase 13, *ADAMTS-5* = metalloproteinase with thrombospondin motif, *Col2a1* = type II collagen, *BCL2* = B-cell lymphoma 2, *SOD-2* = superoxide dismutase 2, *NRF2* = nuclear factor erythroid 2 like 2, *NFKB1* = Nuclear Factor Kappa B Subunit 1 (p50), *RELA* = Proto-Oncogene, NF-KB Subunit (p65), *ACTB* = Actin Beta, *miR* = microRNA, *SNORD-25* = Small Nucleolar RNA, C/D Box 25.
