# Supplementary figures and images for: Synovial Fluid Regulates the Gene Expression of a Pattern of microRNA via the NF-κB Pathway: An In Vitro Study on Human Osteoarthritic Chondrocytes
Source: Int J Mol Sci. 2022 Jul 28;23(15):8334. doi: 10.3390/ijms23158334 (PMC9369022; doi:10.3390/ijms23158334)

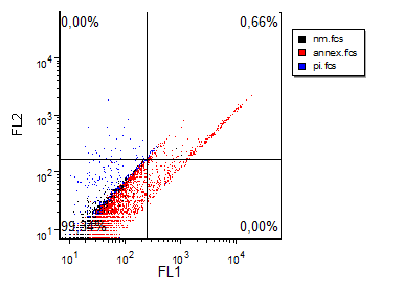

Supplement: Supplementary file 1 [file ijms-23-08334-s001.zip › Figure S1.tif]

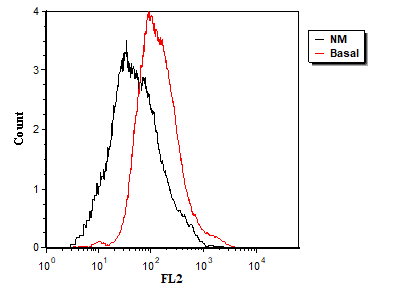

Supplement: Supplementary file 1 [file ijms-23-08334-s001.zip › Figure S2.tif]

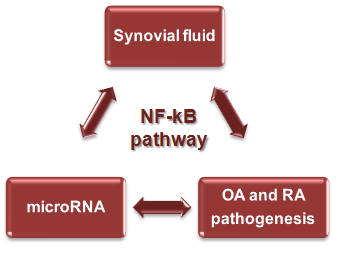

Supplement: Supplementary file 1 [file ijms-23-08334-s001.zip › Figure S3.tif]
